# Effects of Priming Discriminated Experiences on Emotion Recognition Among Asian Americans

**DOI:** 10.3389/fpsyg.2022.797506

**Published:** 2022-03-03

**Authors:** Sophia Chang, Sun-Mee Kang

**Affiliations:** Department of Psychology, California State University, Northridge, CA, United States

**Keywords:** racial discrimination, priming, heightened sensitivity, emotion recognition, Asian American

## Abstract

This study explored the priming effects of discriminated experiences on emotion recognition accuracy of Asian Americans. We hypothesized that when Asian Americans were reminded of discriminated experiences due to their race, they would detect subtle negative emotional expressions on White faces more accurately than would Asian Americans who were primed with a neutral topic. This priming effect was not expected to emerge in detecting negative facial expressions on Asian faces. To test this hypothesis, 108 participants were randomly assigned to one of two conditions: write about their experiences with racial discrimination (experimental) or write about a neutral topic (control). Then, they were given an emotion recognition test consisting of White and Asian faces. The current study found a significant interaction effect of priming condition by target race. When Asian Americans were reminded of discriminated experiences, they displayed heightened sensitivity to negative emotional expressions on White faces, but not to the negative expressions on Asian faces. The implications of these findings were discussed.

## Introduction

In the post-civil rights era, various forms of racism have been identified and studied including aversive racism (Gaertner and Dovidio, 1977, 2005), symbolic racism (Kinder and Sears, 1981), modern racism (McConahay et al., 1981), racial ambivalence (Katz and Hass, 1988), racial resentment (Kinder and Sanders, 1990), subtle prejudice (Pettigrew and Meertens, 1995), and racial microaggression (Sue et al., 2007). Although each version has distinctive features, they share one common view: A person who endorses egalitarian values and considers oneself to be nonprejudiced could hold unconscious negative feelings toward people of color. For example, Whites may show anxiety or discomfort during interactions with Blacks or other marginalized group members (Gaertner and Dovidio, 2005). They may also demonstrate unconscious bias during intergroup interactions (Gaertner and Dovidio, 2005), decision-making processes (Minero and Espinoza, 2016), or public policy endorsements (Sears and Henry, 2003). These studies illuminate the reality of the types of discrimination people of color face. They are confronted by covert and subtle forms of racial prejudice (Sue et al., 2007) on top of the persisting blatant acts of racism (Yoo et al., 2010). Unlike traditional racial discrimination, in which anti-minority sentiments are openly blared, modern-day discrimination can be insidious (Gaertner and Dovidio, 1977, 2005) and even unintentional (Sue et al., 2007).

Experiencing subtle forms of discrimination adds cognitive load to people of color due to attributional ambiguity (Major and Crocker, 1993; Banks and Landau, 2021). People of color often encounter social situations in which they question the motivations behind the negative (at times, even positive) feedback they receive or uncomfortable interpersonal interactions. To resolve the uncertainty, they may spend their cognitive resources to analyze the ambiguous social situations. Such psychological processes may come at a cost of depleted cognitive resources as demonstrated in experimental studies (e.g., Salvatore and Shelton, 2007; Holoien and Shelton, 2012; Espino-Pérez et al., 2018; Banks and Landau, 2021). Particularly, Holoien and Shelton (2012) demonstrated that when participants of color briefly interacted with White participants primed with colorblind approach to diversity, they performed poorly on a cognitive task after the interaction compared to other participants of color interacted with White participants primed with a multicultural approach to diversity. Since White participants primed with colorblind approach to diversity were rated to act more prejudiced than Whites primed with multicultural approach by independent judges in this study, Holoien and Shelton (2012) speculated that interacting with a prejudiced partner would be more cognitively demanding because people of color focus on subtle verbal and nonverbal behavioral cues of discrimination and execute effortful controls on their behaviors accordingly.

It is notable that reading subtle cues of discrimination during social interactions was considered as one of the reasons for cognitive depletion in Holoien and Shelton’s (2012) study. Kunstman and his colleagues (2016) took one step further and argued that when people of color repeatedly experience the attributional ambiguity of Whites’ smile in interracial contexts (i.e., “smiling racism”), they develop sensitivity to the authenticity of Whites’ smiles as a result. The term “smiling racism” was coined by legal scholars to explain Whites’ masking of discrimination in housing and employment with positive behaviors, such as smiles to appear nonprejudiced (Brooks, 1992). Kunstman and his colleagues reasoned that when people of color chronically experience disguised Whites’ smiles, they become suspicious of their genuineness.

To test this idea, Kunstman et al. (2016) examined whether Black participants’ level of suspicion of Whites’ motivation would predict their accuracy to the authenticity of Whites’ smiles. The participants’ level of suspicion was measured using the Suspicion of Motives Index (Major et al., 2013), a self-report questionnaire that evaluates people of color’s suspicion of Whites’ internal and external motives when they demonstrate positive behaviors. As predicted, Black participants with higher levels of suspicion more accurately discriminated between real and fake smiles of White individuals.

The main focus of Kunstman et al.’s study was individual differences in levels of suspicion, meaning that Black individuals with high levels of suspicion of Whites’ motivations tend to be particularly good at reading the authenticity of emotional expressions. However, we suspected that people of color could display heightened sensitivity to Whites’ emotional expressions, independent of individual differences in their levels of suspicion. The heightened sensitivity in this context implies the enhanced accuracy of detecting emotional expressions when people of color are primed by their social situations. For example, if a person of color patronizing a retail store or restaurant has experienced discrimination by White employees, it can be reasonably expected that they will more likely exhibit heightened sensitivity to the facial expressions of White employees the next time they find themselves in similar contexts (e.g., Lee, 2000; Schreer et al., 2009; Bennett et al., 2015). This example suggests that people of color exposed to situations reminding them of past experiences of discrimination may become more sensitive to discriminatory cues.

There is an empirical study supporting this reasoning. Kaiser et al. (2006) demonstrated that when women anticipated interacting with sexist men by given descriptions, they were more attentive toward words associated with sexism on the Stroop task (MacLeod, 1991). Kaiser et al. (2006) reasoned that when women felt their social identity was threatened (such as expecting to interact with sexist men), their attention shifted toward looking for cues that align with their concerns. The result of this study can be extended to other stigmatized group members, such as people of color, who experience discrimination in their daily lives (Sue et al., 2007). When people of color sense discrimination, it may lead them to pay close attention to cues that threaten their racial identity.

### Main Purpose of the Current Study

The current study was conducted to explore this possibility. We speculated that when discriminated experiences were triggered, people of color would be more accurate in recognizing negative emotional expressions of White people compared to people of color who were not reminded of those experiences.^1^ To test this idea, we designed the current study with two priming conditions (discrimination vs. neutral), two intensity levels of expressions (subtle vs. overt), and two racial target groups who displayed negative emotional expressions (White vs. Asian). Due to the lack of prior empirical studies that explored the relationship between experiences of racial discrimination and subtle emotional expressions, we included two different intensities of emotional expressions to test whether the priming effect would be more pronounced in subtle emotional expressions in the current study. The Montreal Set of Facial Displays of Emotion (MSFDE; Beaupré and Hess, 2005) was chosen for this purpose because this is one of the few emotion stimulus sets that offer different levels of intensity of emotional expressions.

Finally, we added two target racial groups to be judged in this study. Since priming would evoke participants to experience two different emotional moods (negative vs. neutral), it was necessary to control for the mood effect on detecting emotional expressions. It has been well documented that under negative mood, information processing tends to be more analytic and detail-oriented (e.g., Schwarz and Clore, 1983; Schwarz, 1990; Isbell and Lair, 2013). To demonstrate that heightened sensitivity to emotional expressions was triggered by primed discriminated experiences, not by negative mood, we needed a comparison condition—emotional expressions of Whites vs. own racial group members. If heightened sensitivity to emotional expressions is solely due to the negative mood triggered by priming, participants should detect emotional expressions better under the discrimination priming condition than the neutral condition regardless of target racial groups. However, if heightened sensitivity to emotional expressions would appear only for White faces, but not for their racial group members, then it would eliminate the alternative explanation of the negative mood effect.

To meet the requirement for adding the control condition of emotions expressed by own racial group members, it was necessary to limit the racial backgrounds of the participants into one race to maintain the complexity of the study design at a manageable level. We chose Asian American participants for two practical reasons: Asian is one of the three racial groups included in the MSFDE (i.e., White, Black, and Asian faces) and the accessibility of this racial group at the study site. It should be noted that even though the current study is primarily based on studies that focused on the Black population, Asian Americans were examined in the present study. This dissociation between the past literature and the current study implies that the findings from the previous studies may not be applied to the current study. Despite this limitation, it would be worthwhile to explore whether Asian Americans would display a similar outcome as Black Americans, given the continued racial discrimination and prejudice that Asian Americans experience.

There was one major hypothesis in the current study. We hypothesized that under the priming condition, Asian American participants would detect subtle negative emotional expressions of White faces more accurately than those in the neutral priming condition would. We expected that this priming effect would not emerge in detecting negative facial expressions of Asian faces. In short, we predicted a 3-way interaction effect of priming (discrimination vs. neutral) by intensity (high vs. low) by target race (Asian vs. White).

## Materials and Methods

### Participants

The current study was conducted with Asian American students who were enrolled in introductory psychology at a large public university on the West Coast. Among self-identified Asian American students who participated in this study, only participants who were born and raised in the United States (*N* = 108) were selected for the final analyses to eliminate the potential confound of different levels of acculturation (Prado et al., 2014) and cultural exposure levels (Sun and Lau, 2018). Out of 108 participants, the priming condition consisted of 61 participants and the control condition of 47 participants.^2^ The sample size for this study was determined based on a power analysis using the G*power program (Faul et al., 2009). Given that there was little previous research directly associated with the current study, we used a conservative estimate of the effect size. The suggested sample size was 46 participants per group (the total sample size of 92) given an estimated effect size value (*f* value) of 0.15 and a statistical power of 0.80.

Participants’ age ranged from 18 to 24 years (*M* = 19.17, *SD* = 1.33) and 62.0% were female (64.2% in the experimental condition and 35.8% in the control condition). The ethnic background of the participants consisted of 24.1% East Asian heritage (Chinese, Japanese, Korean, and Taiwanese), 12.0% South Asian heritage (Bangladeshi, Indian, and Pakistani), and 63.9% Southeast Asian heritage (Cambodian, Filipino, Thai, and Vietnamese). The participants received course credit in exchange for their participation.

### Materials

Participants’ emotion recognition accuracy was measured using the MSFDE (Beaupré and Hess, 2005). The MSFDE provides gray scale photographs of five negative emotions and one positive emotion, totaling six emotions. The facial expressions of anger, disgust, fear, joy, sadness, and shame were depicted by French-Canadian adults residing in Canada and Chinese adults in China who were instructed to pose expressions based on Ekman and Friesen’s (1978) Facial Action Coding System. Although our focus was on detection of negative emotions, we decided to use all six emotions including joy to demonstrate that heightened sensitivity to emotional expressions would be limited to negative emotions but not to positive emotions. In addition, providing the participants with a positive emotion as one of the answers widened their response choice.

The MSFDE also provides four additional levels of intensity of emotion that were created by morphing neutral and original high-intensity emotional expressions, totaling five levels of intensity ranging from 20 to 100, with 20 representing the lowest intensity level and 100 representing the highest intensity level. Among the five intensity levels, two intensity levels (40 and 80) were chosen in the current study to avoid intensity levels that were too ambiguous (such as 20) or too obvious (such as 100). In this study, emotional expressions at intensity level 40 were referred to as low intensity and intensity level 80 were referred to as high intensity. The 96 photos used in this study were evenly divided among two target race groups, two intensity levels of emotional expressions, and six emotions. Thus, four pictures per condition were presented (i.e., 2 target racial groups × 2 intensity levels × 6 emotions × 4 pictures = 96 photos). Half of the 96 photos were female.

The emotion accuracy scores were computed by assigning 1 to correct responses and 0 to incorrect responses. The accuracy scores ranged from 0 to 40 (2 intensity × 5 negative emotions × 4 pictures per emotion = 40 pictures per target race). Each participant received two accuracy scores—one for White faces and one for Asian faces. In the current study, heightened sensitivity was operationally defined as the total accuracy score. The greater the score, the greater the heightened sensitivity.

### Procedure

Each experimental session consisted of 1 to 3 participants in a small room housing three computers with partitions between each computer for privacy. Upon arrival, participants were randomly assigned to the experimental or control condition. Participants were instructed to take approximately 5 minutes to write about the assigned topic that was on the desk. For the experimental condition, they were instructed to write about an incident in which they were treated unfairly because of their race. For the control condition, they were instructed to describe the shoes on their feet (Pennebaker and Beall, 1986; Pennebaker et al., 1988). After the writing was completed, participants were instructed to seal their written paper in an envelope and drop it in a designated box.

Next, a computerized test was administered under seemingly independent studies. In the emotion recognition test, a total set of 96 stimuli was taken from MSFDE and presented in one block. The stimuli were presented in random order on a 15-inch LCD monitor with resolution 1,280 × 1,024 at 60 Hz using the software program, Inquisit 4 [Computer software] (2015). A photo of each face was presented on the center of a black screen for 500 ms. Once the photo disappeared, a list of six emotions (anger, disgust, fear, joy, sadness, and shame) was presented on the screen, from which participants were asked to select one emotion. There was no limit on response time and the scale remained until a selection was made. The entire experimental session took approximately 10–15 minutes to complete.

## Results

### Overview of Data Analyses

The main hypothesis of the current study was that Asian American participants who were reminded of discriminated experiences would detect subtle negative emotional expressions of White faces more accurately than those in the neutral priming condition. In comparison, we hypothesized that the priming effect would not emerge in detecting negative facial expressions of Asian faces. To test this main hypothesis, a 2 (priming condition) × 2 (target race) × 2 (intensity) Mixed ANOVA was conducted on the total accuracy score of five negative emotions. In this Mixed ANOVA, the priming condition was a between-subject factor and the remaining conditions were within-subject factors.

### Preliminary Analyses

Prior to testing the main hypothesis, an initial analysis was conducted to examine the priming effects on emotion recognition with the positive emotion. A 2 (priming condition) × 2 (target race) × 2 (intensity) Mixed ANOVA was conducted with the total accuracy score of recognizing joyful expressions. The results showed that only the main effect of target race was significant, *F*(1, 106) = 24.085, *p* < 0.001, $\eta_{p}^{2}$ =0.185. The recognition accuracy for Asian faces (*M* = 7.57, *SD* = 0.75) was significantly higher than the one for White faces (*M* = 7.16, *SD* = 0.94), implying that Asian American participants in this study recognized joyful expressions on Asian faces significantly better than on White faces. Since there was no significant 2-way interaction effect of priming condition × target race or 3-way interaction effect of priming condition × target race × intensity, these results suggested that heightened sensitivity to emotional expressions of White faces did not emerge with the positive emotion of joy.^3^

### Main Analyses

To test the main hypotheses, another 2 (priming condition) × 2 (target race) × 2 (intensity) Mixed ANOVA was conducted with the total accuracy score of five negative emotions. First, two main effects of target race and intensity were found to be significant in this study. The main effect of target race means that negative emotional expressions of White faces were easier to decode than those of Asian faces, *F*(1, 106) = 76.450, *p* < 0.001, $\eta_{p}^{2}$ = 0.419. It is notable that this effect was opposite to the main effect of target race with the positive emotion found in the preliminary analysis.^4^ There was also a significant main effect of intensity, *F*(1, 106) = 131.660, *p* < 0.001, $\eta_{p}^{2}$ = 0.554, such that emotions in the higher intensity levels were more accurately identified than emotions expressed in the lower intensity levels. As predicted, the main effect of priming was not significant, *F*(1, 106) = 1.370, *p* = 0.245, mainly due to the fact that priming worked only on identifying facial expressions of Whites.

**Figure 1:**
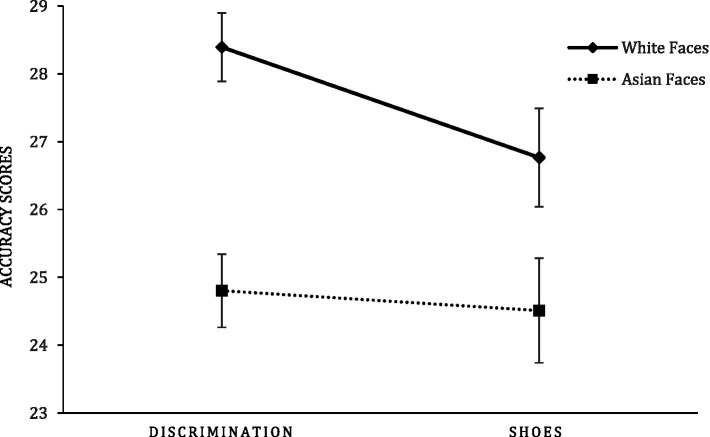
Mean emotion recognition accuracy scores of priming conditions by target race.

Among the interaction effects involving priming condition and target race, the 3-way interaction effects (priming condition × target race × intensity) were not significant. Instead, the 2-way interaction effect of priming condition × target race turned out to be significant, *F*(1, 106) = 3.987, *p* = 0.048, $\eta_{p}^{2}\;$ = 0.036. The means and the standard deviations of emotion accuracy scores of priming condition by target race are displayed in Table 1. As shown in Figure 1, the participants in the experimental condition (*M* = 28.39, *SD* = 3.91) were more accurate in recognizing negative emotional expressions of Whites than the participants in the control condition (*M* = 26.77, *SD* = 4.99), *t*(106) = 1.899, *p* = 0.060, *d* = 0.361. However, there was virtually no difference in recognizing negative emotions of Asians between the experimental condition (*M* = 24.80, *SD* = 4.21) and the control condition (*M* = 24.51, *SD* = 5.28), *t*(106) = 0.320, *p* = 0.749, *d* = 0.062. This significant 2-way interaction effect partially supported our hypothesis that Asian Americans were more accurate at identifying negative emotional expressions of Whites when they were primed with discriminated experiences compared to when primed with the neutral topic.^5^ This priming effect was not pronounced in detecting emotional expressions of Asian faces.

**Table 1:** Means and standard deviations of emotion accuracy scores.

Target race							
	Discrimination (*N* = 61)		Shoes (*N* = 47)		*t* statistics and effect sizes (*df* = 106)		
	*M*	*SD*	*M*	*SD*	*t*	*p*	Cohen’s *d*
Asian Face	24.80	4.21	24.51	5.28	0.320	0.749	0.062
White Face	28.39	3.91	26.77	4.99	1.899	0.060	0.361

*The emotion accuracy scores ranged from 0 to 40 (2 intensity × 5 negative emotions × 4 pictures per emotion = 40 pictures per target race)*.

## Discussion

The main purpose of the current study was to test whether explicitly priming past experiences of racial discrimination would lead Asian Americans to show a heightened sensitivity to subtle negative emotional expressions of White faces more than those in the control priming condition. We also hypothesized that this priming effect would not emerge with Asian faces. Consistent with our hypothesis, Asian Americans who wrote about past experiences of racial discrimination more accurately recognized negative emotional expressions of White faces than Asian Americans who wrote about the neutral topic. In contrast, the participants in both priming conditions did not show any significant differences in detecting negative emotions of Asian faces. The difference in detecting negative emotions of White faces between the two priming conditions implies that reminders of past experiences of discrimination may lead Asian Americans to shift their attention to deciphering emotional expressions on White faces.

The results of the data analyses with positive emotion revealed that heightened sensitivity to emotional expressions on White faces was applied only to negative expressions. Asian Americans in the current study did recognize joyful expressions on Asian faces significantly better than on White faces. Although this finding should be interpreted with caution given that only one positive emotion was used in the current study, divergent sensitivity to facial expressions by emotional valance seems to imply how Asian Americans process emotional information differently depending on the race of the expresser.

To our knowledge, this is one of the first attempts to explore the effects of priming past discriminated experiences on emotion recognition. The main findings of the current study suggest that when reminded of past experiences of discrimination, Asian Americans showed heightened sensitivity to negative emotional expressions of White faces but not of Asian faces. This implies that remembering or thinking about past discriminated experiences may lead people of color to be more cognitively vigilant to negative emotional cues in their social environment.

### Limitations and Future Directions

The current study, however, has a number of limitations that may limit the implications of the current findings. First, it could be argued that heightened sensitivity to negative emotional expressions was simply due to the consequence of experiencing negative feelings, rather than due to remembering discriminated experiences per say. Inducement of negative feelings alone, however, cannot explain the divergent sensitivity to emotion expressions by target race. When participants were reminded of their discriminated experiences, they were not more sensitive to negative expressions of Asian faces compared to the individuals who were primed with neutral cues. If experiencing negative emotions made the participants more sensitive to emotional expressions, they should have shown heightened sensitivity to negative expressions of Asian faces as well.

Another limitation in the current study is the inequivalent nature of the topics across two priming conditions. While the participants in the priming condition were required to write about a social interaction, such as past discriminated experiences, those in the control condition wrote about the shoes on their feet, a topic that did not involve social interactions. It is unclear how the inequivalence of the topics might have influenced the main findings of this study. To address this limitation, future studies may use a topic involving neutral social interactions for the control condition.

Furthermore, it is also possible that being reminded of negative experiences unrelated to discriminated experiences alone can lead to focusing on negative emotions of others. Although negative emotion alone could not explain the interaction effect found in the current study, it would be desirable to replicate the significant interaction effect in a future study with an additional condition of negative experiences unrelated to discriminated experiences.

Emotion stimuli used in this study also introduce another limitation. The MSFDE consists of photos of French-Canadian adults residing in Canada and Chinese adults residing in China. Although the expressers were instructed to pose expressions based on Ekman and Friesen’s (1978) Facial Action Coding System, there may have been nuanced cultural variations. Despite this limitation, the MSFDE was chosen for this study because this stimulus set provides emotional expressions of various races in multiple levels of intensity, allowing us to test for participants’ accuracy in decoding subtle and clearly expressed emotions. However, uncontrolled cultural nuance in emotional expressions may have attributed to participants’ relative difficulty in identifying negative emotional expressions of Asian faces compared to White faces and contributed to the significant main effect of target race.

Another weakness of the current study is that our participants were exclusively Asian American due to the practical reasons that we previously discussed. Moreover, the participants in the current study had a wide range of ethnic backgrounds including Southeast Asian (e.g., Filipino, Vietnamese, Cambodian, Thai, and Indonesian), East Asian (e.g., Chinese, Japanese, Korean, and Taiwanese), and South Asian (e.g., Bangladeshi, Indian, and Pakistani). Since the Asian photos in the MSFDE are taken from Chinese residing in China, the participants with South Asian backgrounds may have been less familiar with the Chinese faces used in this study compared to the participants with East Asian or Southeast Asian backgrounds. However, due to the lack of statistical power, we were not able to test whether the interaction effect of priming condition by target race would hold within each ethnic group. Thus, generalizing our findings to other racial and ethnic groups should be done with caution, and future studies may explore potential differences among these groups.

Related to the characteristics of the participants in this study, it should be mentioned that the current study is not a balanced design study because only Asian participants were recruited. Due to the lack of White participants, the results from the current study should not be interpreted as empirical support for in-group or out-group advantages in emotion recognition mainly because the recognition accuracy obtained from this study is inherently confounded with the “inequivalent test difficulty” of the stimulus sets (Kang and Lau, 2013). The fact that the current study did not use the balanced design could be one of the limitations. However, the balanced design was not pursued from the beginning because exploring the in-group or out-group advantages was not the primary interest of the current study.

Future studies can expand the current study by incorporating methods in which participants experience subtle discrimination in a laboratory experiment, such as using confederates to microaggression against participants prior to taking an emotion recognition test. It would be interesting to explore whether the similar heightened sensitivity to emotional expressions would be observed immediately after people of color experience subtle discrimination. Researchers can also utilize other types of emotion recognition stimuli, such as videos, so that participants are able to select other types of nonverbal cues, such as vocal tones and body language. Lastly, future studies can include participants of various racial and ethnic backgrounds to examine whether the current findings can be replicated with the broader population.

### Closing Remarks

Numerous studies have provided ample evidence of the pernicious effects of racial discrimination, including poorer adjustment and health (Lee, 2005; Gee et al., 2007). However, few have demonstrated the cognitive reactions to primed discriminated experiences. The present empirical study sought to address this gap in the literature by investigating the priming effects of discriminated experiences on emotion recognition accuracy among people of color, specifically Asian Americans. The main findings of the current study suggest that when reminded of past experiences of discrimination, Asian Americans showed heightened sensitivity to negative emotional expressions of White faces. If people of color are reminded of past discriminated experiences, they could be cognitively vigilant to emotional cues, potentially affecting cognitive resources.

## Data Availability Statement

The raw data supporting the conclusions of this article will be made available by the authors.

## Ethics Statement

The studies involving human participants were reviewed and approved by the California State University, Northridge Internal Review Board. Written informed consent for participation was not required for this study in accordance with the national legislation and the institutional requirements.

## Author Contributions

SC proposed the research idea, programmed the emotion recognition test, collected the data, conducted the initial data analyses, and drafted the manuscript. SMK formulated the overall study design, oversaw all data collection, conducted the final data analyses and wrote the results section, and provided critical revisions.

## Conflict of Interest

The authors declare that the research was conducted in the absence of any commercial or financial relationships that could be construed as a potential conflict of interest.

## Publisher’s Note

All claims expressed in this article are solely those of the authors and do not necessarily represent those of their affiliated organizations, or those of the publisher, the editors and the reviewers. Any product that may be evaluated in this article, or claim that may be made by its manufacturer, is not guaranteed or endorsed by the publisher.

## Footnotes

^1^Although Kunstman and his colleagues (2016) tested sensitivity to the authenticity of Whites’ smiles, we limited our hypothesis to negative emotions only because we questioned whether a fake smile with discriminatory attitudes could be considered a positive expression. In addition, fake smile was not one of the emotional expressions displayed in the study stimuli we used in the current study.

^2^While we aimed to balance the number of participants in both conditions, when we filtered out the participants not born in the United States, the sample sizes of the two conditions became uneven. The outcomes of the current study might have been compromised by its imbalanced sample sizes.

^3^The outcomes of the preliminary analyses should be interpreted with caution because they were based on a single emotion, which made this measure less reliable than the one for negative emotion. It should also be mentioned that identifying the correct negative emotion out of five choices was more challenging than recognizing the single positive emotion in the test. This may have influenced the outcomes of the current study because it required additional cognitive resources to identify the correct negative emotions.

^4^There is one study (BeauprÃ© and Hess, 2005) that reported the recognition accuracy of six emotions using the Montreal Set of Facial Displays of Emotion and Asian participants. Although it is not easy to compare the findings from the current study with the reports from BeauprÃ© and Hess (2005) mainly due to considerable differences in the study procedure and the characteristics of study participants, they reported that recent Chinese immigrants residing in Canada showed better recognition accuracy of Chinese faces over White faces in general regardless of emotion valance.

^5^Most of the participants in the priming condition wrote about their general experiences of racism over the years, such as being told racist jokes, being called a racial slur, or having received mistreatment due to race. Out of 61 participants, three specified the race of the perpetrators (all reported as being White) and two stated living in a predominately White community and experiencing various forms of racism throughout their lives.
